# Occlusion and Temporomandibular Disorders: A Scoping Review

**DOI:** 10.3390/medicina61050791

**Published:** 2025-04-24

**Authors:** Laurențiu Pascu, Raul-Samuel Haiduc, Oana Almășan, Daniel-Corneliu Leucuța

**Affiliations:** 1 Department of Prosthetic Dentistry and Dental Materials, Iuliu Hațieganu University of Medicine and Pharmacy, 32 Clinicilor Street, 400006 Cluj-Napoca, Romania; 2 Department of Medical Informatics and Biostatistics, Iuliu Hațieganu University of Medicine and Pharmacy, 400349 Cluj-Napoca, Romania

**Keywords:** temporomandibular disorders, disc displacement, muscle disorder, osteoarthritis, occlusion, occlusal disharmony, malocclusion, orofacial pain, parafunction

## Abstract

*Background and Objectives*: The occlusal–temporomandibular disorder (TMD) relation is a contentious issue in dentistry to date. This scoping review’s purpose was to map the existing literature on occlusal abnormalities and their potential role in the development and progression of TMD. *Materials and Methods*: A search in PubMed, Scopus, Cochrane Library, Embase, Lippincott, Medknow, and ClinicalKey was conducted. Articles researching the relationship between TMD and occlusion have been selected. A narrative data synthesis was conducted to chart and summarize the main findings from the included studies. *Results*: A total of 29 articles were included in this review. These studies confirm that angle class II and angle class III malocclusions, deep bite, and crossbite have a high prevalence of symptoms of TMD, including mandibular deviation, arthritic pain, and tenderness of the muscles. Malocclusion, edentulous spaces, and a reduced vertical dimension of occlusion (VDO) also contribute to the severity of TMD, most prominently in older adults. TMD is also seen with high prevalence in females, with a female-to-male ratio of 2:1 to 20:1, according to studies. Bruxism, premature occlusal contacts, and occlusal interferences also contribute towards symptoms of TMD, in agreement with multiple facets of the disorder. *Conclusions*: Occlusal abnormalities have a significant association with TMD, but causality cannot be established with most observational studies. This review emphasizes the need for early occlusal examination and intervention to reduce TMD risk.

## 1. Introduction

Temporomandibular disorder (TMD) is a general term for a collection of neuromuscular and musculoskeletal pathologies that include temporomandibular joint (TMJ), masticatory muscles, and supporting structures [1]. Although prevalence data vary, it is believed that between 3.2 and 15% or 17.6% of individuals suffer with TMD, with women between the ages of 20 and 40 having a greater frequency, approximatively 2.1 times more than men [2,3]. TMD peaks during the reproductive years in women [4]. The prevalence of TMD in young people and adolescents ranges from 20% to 60% [5].

The etiology of TMD is multi-factorial, both in terms of biological, psychological, and environmental factors, and it often overlaps with other syndromes, including headaches, fibromyalgia, and syndromes of chronic pain [6,7]. Risk factors for temporomandibular disorders were female gender, depression, anxiety, stress, sleep disturbances, obstructive sleep apnea, headache, migraine, and other chronic pains [8,9]. In adolescence, the etiology of TMD can include dental anomalies, bad habits, growth abnormalities, and stress [10].

The symptoms of TMD include pain in the TMJ and masticatory muscles [11], joint sounds (crepitus and clicking), locking [12,13,14] restrictions on jaw mobility [15,16,17], and disc displacement (DD) [18,19,20,21].

The diagnosis of TMD is a complex process that requires a comprehensive evaluation of the TMJ signs and symptoms, acquired through clinical tests, examinations, and medical image analyses [22]. Imaging modalities for the evaluation of TMD include panoramic imaging, arthrography, computed tomography (CT) magnetic resonance imaging (MRI), and cone beam computed tomography (CBCT) [23,24,25,26]. Temporomandibular joint osteoarthritis is a chronic degenerative disease that can result from TMD, TMJ disc displacement, trauma, functional overload, and developmental anomalies [27]. It affects the cartilage, the subchondral bones, the synovial membrane, and other hard and soft tissues [28], with manifestations such as bone remodeling and cartilage damage [29].

The equilibrium of the masticatory system can be disrupted by a variety of factors; however, one factor generates significant debate. Occlusal disharmonies are recognized as an etiological factor, but the extent and manner in which they contribute to TMD remain unclear [30]. Occlusal dynamics affect multiple interfaces, including the teeth, periodontium, masticatory muscles, and the temporomandibular joint. The mechanical stress exerted on these interfaces can compromise their integrity [31]. Occlusal interferences and occlusal dysfunctions can lead to orthopedic joint instability and the hyperactivity of the masticatory muscles, which can result in TMD [32].

The relationship between occlusion and TMD remains ambiguous, which has resulted in conflicting study findings [33,34]. The clinical signs of TMD have been correlated with certain occlusal factors and parafunctional habits: occlusal variables that increase the likelihood of different signs of TMD may be interferences in centric relation (CR), discrepancy between midline ≥ 2 mm, ≤10 contacts during maximum intercuspation, interferences on the non-working (passive) side and overjet ≥ 5 mm [35], parafunctional habits (grinding and clenching), occlusal tooth wear [36,37,38], and unilateral posterior crossbite [38,39,40]. TMD has been also associated with deep overbite [35,41], posterior scissor bite, angle class II malocclusion [35,42], anterior open bite [43], unstable occlusion [44], neck posture [45], and excessive overjet [46].

Functional malocclusions have a greater influence on cranio-mandibular dysfunction than morphologic malocclusions [47]. To differentiate TMD patients from those who function normally, however, no unique occlusal characteristic was feasible [48,49]. A biological organism will continuously adapt to various morphologic elements until equilibrium is achieved [34]. Changes in head posture impact the balance of occlusal contacts [34].

Previous research in occlusion and TMD varies and tends to be based on individual variables or specific inclusion criteria that limit generalizability [50]. TMD classification variability and methodological inconsistencies are significant contributors to incompatible findings [51]. The goal of this scoping review was to map the literature and clarify the role of occlusion in the development of TMD. This study’s objectives were to find a relationship between temporomandibular dysfunction and occlusal balance and understand how the occlusion influences temporomandibular joint pathology and/or masticatory and cervical muscle symptoms.

## 2. Materials and Methods

This scoping review followed the Preferred Reporting Items for Systematic Reviews and Meta-Analyses extension for Scoping Reviews (PRISMA-ScR) Checklist [52].

### 2.1. Eligibility Criteria

All studies focusing on the relationship between TMD and occlusion were included in this review. We excluded publications such as case reports, systematic reviews, editorials, animal studies, and conference abstracts.

### 2.2. Information Sources

Four databases were accessed for study identification: PubMed (US National Library of Medicine National Institutes of Health), Scopus, Cochrane Database, and Embase, until October 2023.

### 2.3. Search Strategy

The search strategy was based on the following keywords: (temporomandibular disorders OR disc displacement OR muscle disorders OR capsulitis OR osteoarthritis) AND (centric relation OR occlusion OR long centric OR anterior guidance OR occlusal disease OR tooth wear OR tooth ache OR tooth mobility OR malocclusion OR cavities). This strategy was further adapted for each database (Table 1). Synonyms and thesaurus terms (MeSH) were used to increase the sensitivity of the search strategy.

### 2.4. Selection of Sources of Evidence

The screening and selection of articles for inclusion in this review were carried out in the Rayyan software [53]. The eligibility requirements for every selected paper were evaluated separately by two independent experts. The authors (R.-S.H.) and (L.P.) performed the screening of articles based on titles and abstracts. In the case of disagreement, the selection was based on erring toward inclusion. Then, the selection based on the full text of the articles was performed by the authors (R.-S.H. and L.P.). Disagreements were solved by discussion.

### 2.5. Data Items

The following features of the study were extracted from the articles following selection criteria: country, study type, number of participants’ age, reported parameters, and conclusions.

## 3. Results

### 3.1. Study Selection

**Figure 1:**
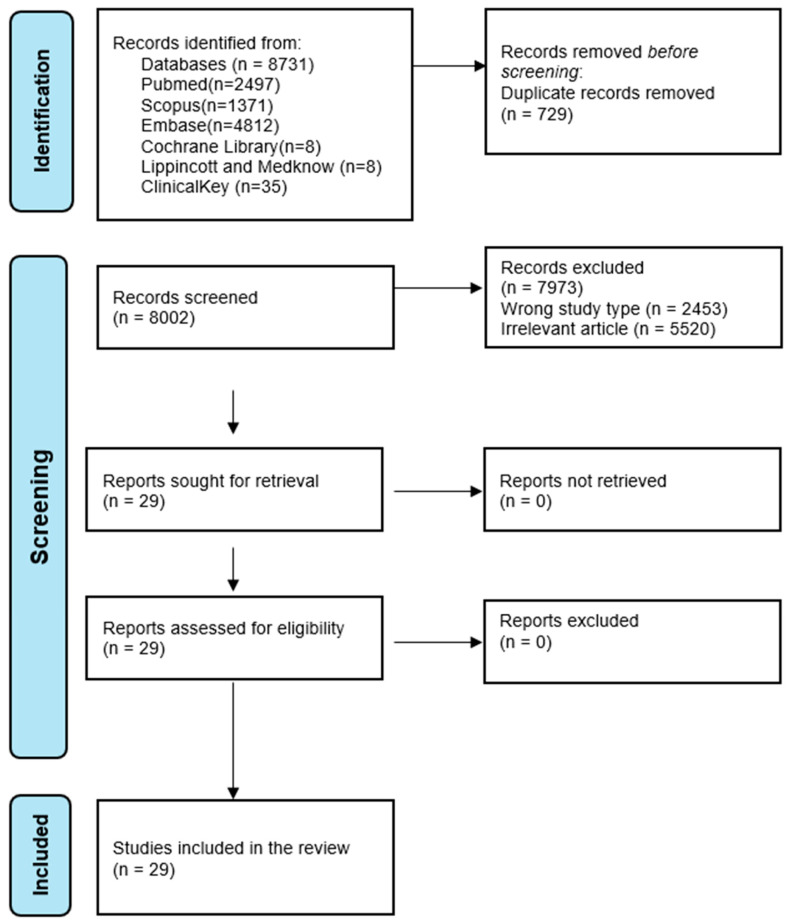
Flowchart presenting the identification, screening, and inclusion of study articles.

Within the databases, the search strategies provided a total of 8731 publications (PubMed (*n* = 2497), Scopus (*n* = 1371), Embase (*n* = 4812), Cochrane Library (*n* = 8), Lippincott and Medknow (*n* = 8), and ClinicalKey (*n* = 35)) (Figure 1). All these results were entered into Rayyan and 729 duplicates were found and removed. The remaining 8002 studies were screened against the inclusion and exclusion criteria. A total of 2453 studies were excluded because the type of study (reviews, case reports, editorials, conference abstracts, letters to editor) did not meet the criteria, and a further 5520 were excluded because they were irrelevant (articles were excluded if they were non-human studies, not focused on the relationship between occlusal factors and temporomandibular disorders (TMD), did not report relevant occlusal variables or TMD outcomes, focused solely on treatment without discussing occlusion). Finally, 29 studies were included in this review.

### 3.2. Study Characteristics

Research has been carried out throughout North America, South America, Europe, and Asia, with a notable number of studies coming from the US, Brazil, and Japan (Table 2). Although the research designs vary, observational studies, especially cross-sectional and comparative studies, predominate; however, some studies have an unspecified design. These studies range widely in terms of the number of participants, from small-scale studies with less than 50 participants to studies with more than 4000 participants. These studies’ reported parameters include a broad range of TMD-related clinical and diagnostic characteristics. Several studies examine malocclusion (M), muscular pain (MP), occlusal abnormalities (OA), and the connection between TMD and gender (AG). Other studies center on certain skeletal and dental characteristics, such as the existence of edentulous spaces (E), angle classification (AC), and the vertical dimension of occlusion (VDO). Studies additionally investigated how TMD affects temporomandibular joint conditions, especially how it relates to osteoarthritis.

The characteristics of the selected studies are presented in Table 2.

### 3.3. TMJ Involvement Correlated with Posterior Edentulism and VDO Changes

A number of studies showed an important relation between VDO changes, edentulous spaces, and TMD (Table 3). While Nguyen (2017) [79] showed a higher incidence of posterior edentulous spaces, especially in the mandible, among TMD patients, Wang (2009) [81] found that TMD susceptibility increased with the number of edentulous quadrants and increasing age. Similarly, Mélou (2024) [35] found that those with untreated edentulous spaces had a higher frequency of TMD.

Additionally, decreases in VDO appears to increase the severity of TMD. According to Joy (2021) [54], more severe TMD, particularly in elderly people, was linked to decreased VDO and increasing interincisal angles. Malheiros (2016) [58] also found an association between TMD and edentulous spaces.

### 3.4. The Influence of Occlusal Abnormalities on the Temporomandibular Joint

A number of studies specify how occlusal abnormalities influence TMD (Table 4). According to Mélou, 2024 [35], laterotrusive interferences and an anterior overbite higher than 4 mm increase the incidence of TMD. According to a number of studies, including those by Barrera-Mora, 2012 [60]; Sonnesen, 1998 [78]; and Al-Hadi, 1993 [57], increased overjet is an important risk factor for TMD. The development of TMD is influenced by occlusal discrepancies and interferences. Raustia (1995) [76] and Pullinger, (1988) [74] also associated asymmetrical sliding between CR and MI, along with irregularities in mandibular movements to joint dysfunction. Seligman (1989) [59] also stated that asymmetric anterior guidance and larger MI-CR discrepancies are due to osteoarthritis. Symptoms of TMD are intensified by functional behavior and premature occlusal interference. Ohmori, in 2013 [71], mentioned that premature contacts directly relate to one of the main etiological factors. Ozaki, 1990 [61], also reported that dental wear, bruxism, and unilateral mastication were the common complaints of TMD patients. Further, Pahkala, 2002 [72] reported that the occlusal interferences were related to clicking and mandible deviations, along with muscle pain.

### 3.5. The Influence of Occlusal Abnormalities and Malocclusions on the Temporomandibular Joint

A number of studies (Table 5) have identified occlusal abnormalities and malocclusions as contributing to the development of TMD. Barrera-Mora (2011) [60] identified that angle class II malocclusion and open bite were associated with ligamentous hyperlaxity in the TMJ, while crossbite was considered a cause of TMD. A number of research studies also demonstrate similar results, including those by Perrotta (2019) [73], Pullinger (1988, 1991) [74,75], and Sonnesen (1998) [63]; their findings indicate open bite and unilateral or bilateral crossbite strongly correspond to joint dysfunction, again reinforcing the occlusal discrepancies related to TMD etiology. Malocclusion classes seem to variably affect TMJ function. According to Pahkala, 2002 [72], in cases of malocclusion, individuals with angle class III are at a risk of mandibular hypomobility and those with angle class II/1 malocclusion have a predisposition to mandibular hypermobility. Seligman, 1989 [59], and Pullinger, 1988 [74], also established an increased risk of joint tenderness and osteoarthritis in cases of angle class II/1 malocclusion as compared to angle class I and postulated that sagittal discrepancies in occlusion may be contributory to TMJ instability. Other occlusal features have also been implicated in the risk of developing TMD, including deep bite, overbite, and overjet. Manfredini, 2010 [69], found that an overjet greater than 4 mm, overbite greater than 5 mm, and bruxism were significant risk factors in TMD. In 2008 [63], Sonnesen also related overbite to bruxism, further underlining the interaction between occlusal morphology and parafunction in the development of TMD.

### 3.6. Relationship Between Joint Impairment and Angle Class

The observations in Table 6 illustrate the relationship between angle classification and joint impairment. According to Mélou (2024) [35], subjects with angle class I have a low level of TMD prevalence. In contrast, a variety of studies have concluded a relation between angle class II malocclusion and symptoms of TMD. Sonnesen (1998) [78] concluded a relation between angle class II and TMD, and Riolo (1987) [77] concluded that subjects with an angle class II half cusp exhibited a high susceptibility for developing symptoms of joint pain, joint clicking, and myofascial pain. In addition, de Paiva Bertoli (2018) [64] concluded that subjects with angle class II and angle class III malocclusions exhibited susceptibility to painful myofascial symptoms. As discussed by Pahkala (2002) [72], subjects with TMD showed angle class III.

### 3.7. Relationship Between Masticatory Muscle Pain and Contracture

The observations in Table 7 present muscle pain and its relation to contracture. Zúñiga-Herrera (2022) [55] documented that pain in the orofacial region is related to dento-maxillary malocclusions, restriction in mouth opening, and depression, with an accentuation of its multi-factorial character. Likewise, similarly, de Paiva Bertoli (2018) [64] documented that skeletal class II and III malocclusions present a larger proportion of masticatory pain with skeletal class I, reinforcing occlusal relations and its relation to muscle dysfunction. Sonnesen L (2008) [78] and Bindayel N (2018) [66] both documented deep bite and its relation to pain, with the latter documenting that crossbite is a risk factor for masticatory and orofacial pain.

A range of studies have confirmed myalgia to be a prevalent sign in TMD subjects. In a report by Ai (1992) [56], 96% of TMD subjects present with palpation pain in both joints and masticatory muscles, with changing myalgia patterns to disharmony in masticatory muscles. Ozaki (1990) [61] observed 64.78% of TMD subjects with pain on palpation of TMJ and masticatory muscles. Sixty percent of subjects with TMD exhibited myalgia in masticatory muscles, according to a report by Tsolka (1995) [80]. Pahkala (2002) [72] observed patients with symptoms including sounds, spasms in masticatory muscles, and deviation of mandible during mandible opening, indicative of TMD with progressive impairment in masticatory muscles.

### 3.8. Degree of Joint Damage in Females

The observations in Table 8 show the severity of impairment in joints in females. Various studies present a much larger female-to-male ratio of TMD prevalence. As per a report by Wang (2009) [81], there is a female-to-male ratio of 2:1. Ozaki (1990) [61] reported a 3.4:1 female-to-male ratio, with an equivalent 3.44:1 female-to-male ratio in a large cohort of 4528 subjects, according to Cooper B (2006) [62]. According to a report by Joy, a very large female-to-male imbalance was observed, with a 20:1 proportion, which is a strong reflection that TMD disproportionately involves females (2019) [54].

A variety of studies confirm not only the higher prevalence of TMD but also present specific symptoms concerning joint dysfunction. The female gender, according to Pahkala R (2002) [72], is most closely associated with TMD-specific symptoms and signs. According to Zúñiga-Herrera (2022) [55], females have a high risk of developing pain in muscles but with no restriction in mouth opening. Barrera-Mora (2011) [60] concluded that females have a high risk for developing the hyperlaxity of joints, mandibular deviation with opening, and the presence of sounds in joints, indicative of increased joint instability and dysfunction when compared with males.

## 4. Discussion

Our scoping review managed to extract a synthesis of the existing literature on TMD and various parameters. The studies included in this review represent a range of geographical locations, and thus include subjects with various social, cultural, and geographical backgrounds, possibly allowing for controlling for genetic, environmental, and behavior-related factors that could affect the disorder. Study types were observational (comparative, prospective, retrospective, cross-sectional, and case–control studies). Publications have linked a range of causative factors for temporomandibular dysfunction, including a change in vertical occlusal dimension and posterior edentulous spaces, occlusal abnormalities, dento-maxillary malocclusions, angle classes, pain, spasms, and the female gender.

### 4.1. TMJ Involvement Correlated with Posterior Edentulism and VDO Changes

The following inferences can be drawn from the literature: the loss of posterior support and secondary occlusal vertical dimension shifts contribute to the development and progression of TMD [79,81]. The loss of occlusal stability in untreated edentulous spaces can produce compensatory neuromuscular adaptations, increased loading of joints, and occlusal disharmony, all of them proven to be TMD contributing factors [35]. The progression of TMD with compromised VDO underlines occlusal collapse in a biomechanical manner, with aging groups at a high risk [54]. Based on these observations, early interventions in terms of prosthetics for restoration of edentulous spaces and maintaining a functional VDO could be critical in preventing and controlling symptoms of TMD. Long-term occlusal rehabilitation and its role in preventing the progression of TMD have to be addressed in future studies.

### 4.2. The Influence of Occlusal Abnormalities on the Temporomandibular Joint

The identified papers show the important role of occlusal stability in TMJ functionality. Occlusal discrepancies such as increased overjet, overbite, and premature contacts have been seen to cause the excessive loading of joints and changed mandibular function, all contributing to increased symptoms of TMD [35,57,60,78]. Asymmetric mandibular movements and mandibular occlusal contact discrepancies (MI-CR) may be due to osteoarthritis, and it can be postulated that long-term occlusal disharmony can cause disease in joints [59]. Bruxism, dental wear, and unilateral mastication have a strong relationship with TMD [61]. Parafunction can cause and maintain disease in joints and muscles and cause occlusal disharmony. Based on these observations, occlusal realignment, functional rehabilitation, and early intervention for occlusal interferences can have a significant role in preventing and managing TMD.

### 4.3. The Influence of Occlusal Abnormalities and Malocclusions on the Temporomandibular Joint

The selected papers suggest malocclusions and occlusal abnormalities have a significant contribution to TMJ dysfunction through a disruption in mandibular movements and an increase in joint tension. Open bite and crossbite have been observed to affect joint stability, most likely through a lack of occlusal support and secondary adaptations in joints, ligaments, and muscles [63,73,74,75]. Malocclusion variation in TMJ function between angle classes shows discrepancies in the sagittal plane have an impact on joint mobility, with hypermobility in cases of angle class II/1 and hypomobility in cases of angle class III, and could lead to long-term destructive processes [72]. Increased overjet and overbite and bruxism have been observed to contribute to TMD occurrence [69]. Occlusal discrepancies and malocclusion must be resolved in an attempt to prevent or at least minimize TMD development. Untreated malocclusions could predispose an individual to long-term secondary muscular and joint pain. Occlusal balancing, occlusal adjustment, and/or restoration could become a critical consideration in TMJ maintenance and function.

### 4.4. Relationship Between Joint Impairment and Angle Class

The literature reveals that occlusal classifications make a significant etiological contribution towards temporomandibular disorder development, with specific malocclusion types predisposing subjects towards pathology in joints and muscles. A low prevalence of TMD in angle class I subjects reveals a role for a harmonious occlusion in TMJ stability [35], in contrast with increased susceptibility in angle class II subjects towards joint pain, sounds, and myofascial pain [77,78]; these results suggest that mandibular retrusion and the changed position of the condylar head produce a strain in occlusal disharmony in angle class II subjects. Angle class II and III malocclusions both have a relation with painful myofascial symptoms [64]. Discrepancies in both sagittal and vertical dimensions contribute towards both joint instability and strain in the muscles and reveal a contribution of malocclusion and occlusal imbalance in TMJ pathology.

### 4.5. Relationship Between Masticatory Muscle Pain and Contracture

These studies validate significant occlusal discrepancies, muscle dysfunction, and TMD pain, supporting the multi-faceted etiology of the disorder. The association between dento-maxillary malocclusions and orofacial pain [55] and between occlusal disharmony and overload in masticatory muscles validates changed occlusal function in the causation of myalgia and restriction in function. The association between increased masticatory myalgia in skeletal class II and III malocclusions [64] and in deep bite and crossbite [66,78] validates occlusal disharmony in causing strain in muscles. A high prevalence of palpation myalgia in TMD subjects [56,61,80] validates the significant muscular contribution in the disorder, with variable groups of muscles having variable types of myalgia in relation to occlusal disharmony and functional adaptations. Muscle spasm, mandibular deviation, and progressive impairment in masticatory muscles validate the long-term contribution of muscle dysfunction in increased symptoms in TMD [72], with long-term impact for joint stability. All these studies validate the use of multidimensional evaluations in TMD, taking both occlusal and muscular factors into consideration, and early intervention programs in occlusal correction, relaxation in muscles, and behavior therapy for occlusal disharmony, myalgia, and improvement in function.

### 4.6. Degree of Joint Damage in Females

That female-to-male ratios have consistently been documented in a variety of studies at a level suggesting a gender predisposition, possibly hormonal, anatomical, or functional in etiology. The female–male prevalence for TMD span in a range between 2:1 [81] and an extreme 20:1 [54]. Female-related symptoms, such as mandibular deviation during opening and joint sounds, which are present in most cases [60], point towards a role for such factors in TMD pathophysiology. Muscle pain and the female gender, even in cases with no restriction in mouth opening, underline neuromuscular factors in TMD pathophysiology. Females have a high likelihood of reporting individual symptoms referable to joints, such as hyperlaxity and deviation. All such observations point towards a gender-related consideration in diagnosing and managing TMD, with a consideration for a role for estrogen fluctuations, ligamentous laxity, and variation in female gender-related perception of pain in female patients.

### 4.7. Comparison with Published Reviews

Recent systematic reviews, such as those conducted by Manfredini et al. (2017) [82] and Trivedi et al. (2022) [83], are in agreement with our scoping review in concluding that occlusal considerations alone are not the only associated factor of TMD. While our review shows associations between TMD symptoms and occlusion type (deep bite, crossbite, and angle class II and III relationships), Manfredini et al. identified only mediotrusive interferences as being related to TMD, and in this instance, causation could not be established [82]. Similarly, in a meta-analysis, Trivedi et al. identified an increased prevalence in TMD sufferers for deep overbite and class II/III relationships, but recognized limitations in making definitive conclusions based upon the fact that the investigations were heterogeneous [83]. Lekaviciute et al. (2024) [84] additionally emphasized tooth loss and bruxism as factors associated with TMD, underlying the multi-factorial nature of the disorder, which we have identified in our findings as well. The biggest shortfall in each review, including ours, is still the lack of longitudinal and interventional research to be able to definitively state if occlusal factors have an impact upon TMD occurrence.

### 4.8. Limitations

A significant limitation is that the studies included in the analysis have a variation in terms of study design, sample, and methodology. Discrepancies in terms of reporting, measurement, and diagnostic criteria can arise, and direct comparisons are not possible. Variability can hinder generalizability and make it unfeasible to make definite statements about causality between occlusal factors and TMD.

Another limitation of this review is that the included studies have an observational nature, and causality between occlusal factors and TMD cannot therefore be confirmed.

Confounders, which are a common issue in observational studies, like parafunctional behavior (bruxism), can have a profound influence upon the occlusion/TMD association as a mediator or as a separate risk factor. Unless they are corrected for, they can distort and not accurately reflect the actual influence of occlusion on TMD and can yield over- or under-estimation.

Whereas the extraction of information focused on key study parameters, an omission of a full evaluation for bias in a review entails that the defects in the methodologies of individual studies could not have been effectively addressed. As mapping current studies is the purpose of this scoping review and not an appraisal of the quality of the evidence, a risk of bias analysis was not performed.

### 4.9. Study Strengths

First, this study adheres to the PRISMA-ScR, with a systemic and transparent selection, extraction of information, and reporting. By searching in a range of key databases (PubMed, Scopus, Cochrane Library, and Embase), a high proportion of relevant studies is captured. By using a search strategy with MeSH terms and synonyms, an increased search sensitivity is achieved, and a high level of issue coverage is assured. Having a variety of types of studies allows for a full mapping of current studies, trends, gaps, and important findings regarding occlusion and TMD. With a variety of occlusal factors, such as malocclusions, overjet, overbite, and occlusal interferences, a holistic view of occlusal factors in developing TMD was considered. One strength the review holds is its novelty, as the literature on occlusal variables and TMD has rarely been charted by other studies, providing and comprehensive summary of the multi-faceted relation.

### 4.10. Future Research

In the future, more research should be aimed at performing well-designed prospective and interventional studies with established occlusal parameters and TMD classification diagnostic criteria. There is a need for better methodological consistency, including clear definitions, uniform measurement tools, and controls for confounding variables such as bruxism and gender. Longitudinal studies should provide insight into the temporal interaction between occlusal defects and the development or worsening of TMD, an essential missing piece in the current evidence.

This review forms part of an ongoing discussion in occlusion in TMD and is beneficial for clinicians and researchers in providing useful information. By providing an overview of current studies, it forms a basis for future systemic reviews and clinical studies, and aids in formulating more specific therapeutic and diagnostic approaches for TMD management.

## 5. Conclusions

This scoping review emphasizes the complex occlusal–temporomandibular disorder relation, with a variety of occlusal abnormalities, such as malocclusions, overjet, overbite, crossbite, and occlusal interferences, having a direct relation with masticatory pain and joint dysfunction. Angle class I occlusion seems to have a lesser incidence of TMD, but angle class II and angle class III malocclusions, deep bites, and edentulous spaces have a predisposition towards symptoms of TMD, such as mandibular deviation, masticatory muscle pain, and tenderness. In addition, this study confirms multi-faceted character of TMD, with occlusal factors, combined with parafunction habits and systemic factors, such as aging and gender predisposition. Causality between occlusion and TMD could not be established based on observational studies.

## Figures and Tables

**Table 1:** Search strategy.

Database	Search Strategy
PubMed	(“temporomandibular disorders” [All Fields] OR “Disc Displacement” [All Fields] OR “Muscle Disorders” [All Fields] OR (“bursitis” [MeSH Terms] OR “bursitis” [All Fields] OR “capsulitis” [All Fields]) OR (“osteoarthritis” [MeSH Terms] OR “osteoarthritis” [All Fields] OR “osteoarthritides” [All Fields])) AND (“centric relation” [All Fields] OR (“dental occlusion” [MeSH Terms] OR (“dental” [All Fields] AND “occlusion” [All Fields]) OR “dental occlusion” [All Fields] OR “occlusion” [All Fields] OR “occlused” [All Fields] OR “occlusions” [All Fields] OR “occlusive” [All Fields] OR “occlusives” [All Fields]) OR “long centric” [All Fields] OR “anterior guidance” [All Fields] OR “occlusal disease” [All Fields] OR “tooth wear” [All Fields] OR “tooth ache” [All Fields] OR “tooth mobility” [All Fields] OR (“malocclusal” [All Fields] OR “malocclusion” [MeSH Terms] OR “malocclusion” [All Fields] OR “malocclusions” [All Fields] OR “malocclusive” [All Fields]) OR (“cavity s” [All Fields] OR “dental caries” [MeSH Terms] OR (“dental” [All Fields] AND “caries” [All Fields]) OR “dental caries” [All Fields] OR “cavities” [All Fields] OR “cavity” [All Fields]))
Scopus	(“temporomandibular disorders” or “Disc Displacement” or “Muscle Disorders” or capsulitis or osteoarthritis) AND (“centric relation” or occlusion or “long centric” or “anterior guidance” or “occlusal disease” or “tooth wear” or “tooth ache” or “tooth mobility” or malocclusion or cavities)
Cochrane Library	(“temporomandibular disorders” OR “Disc Displacement” OR “Muscle Disorders” OR capsulitis OR osteoarthritis) AND (“centric relation” OR occlusion OR “long centric” OR “anterior guidance” OR “occlusal disease” OR “tooth wear” OR “tooth ache” OR “tooth mobility” OR “malocclusion” OR “cavities”)
Lippincot și Medknow	(“temporomandibular disorders” or “Disc Displacement” or “Muscle Disorders” or capsulitis or osteoarthritis) AND (“centric relation” or occlusion or “long centric” or “anterior guidance” or “occlusal disease” or “tooth wear” or “tooth ache” or “tooth mobility” or malocclusion or cavities)
Embase	(‘temporomandibular joint disorder’/exp OR ‘disc displacement’/exp OR ‘muscle disorder’/exp OR capsulitis OR ‘osteoarthritis’/exp) AND (‘centric relation’ OR ‘occlusion’/exp OR ‘long centric’ OR ‘anterior guidance’ OR ‘occlusal disease’ OR ‘tooth wear’/exp OR ‘toothache’/exp OR ‘tooth mobility’/exp OR ‘malocclusion’/exp OR ‘dental caries’/exp)
ClinicalKey	(“temporomandibular disorders” OR “Disc Displacement” OR “Muscle Disorders” OR capsulitis OR osteoarthritis) AND (occlusion OR “occlusal disease” OR “tooth wear” OR “tooth mobility” OR malocclusion)

**Table 2:** Characteristics of included studies.

Article	Country	Number of Participants	Study Type	Age (Years) Mean/Range	TMD Assessment	Reported Parameters
Joy, 2021 [54]	India	160	CS	17–32	n.a.	TMD/VDO/GS
Mélou, 2024 [35]	France	57	CS	CaG: 38.7/CtG: 45.7	RDC/TMD	TMD/OA/TL/AC
Zúñiga-Herrera, 2022 [55]	Mexico	327	CS	24	RDC/TMD	TMD/MP/AG
Ai, 1992 [56]	Japan	210	CS		n.a.	TMD/MP
Al-Hadi, 1993 [57]	Iraq	600	CS	18–22	n.a.	TMD/OA
Malheiros, 2016 [58]	Brazil	150	CS	18–60	FAI	TMD/E
Seligman, 1989 [59]	US	418	CS		n.a.	TMD/OA/M
Barrera-Mora, 2012 [60]	Spain	162	CS	40	Roc, HI	TMD/M/OA/AC/AG
Ozaki, 1990 [61]	Japan	71	CS		n.a.	TMD/MP/AG
Cooper, 2006 [62]	US	4528	CS	11–70	n.a.	TMD/MP/AG
Sonnesen, 2008 [63]	Denmark	60	CS	20–60	RDC/TMD	TMD/M/MP
de Paiva Bertoli, 2018 [64]	Brazil	800	CS	10–14	RDC/TMD	TMD/MP/AC
Filho, 2015 [65]	Brazil	73	CS		RDC/TMD	TMD/M
Bindayel N, 2018 [66]	Saudi Arabia	437	CS	29.41	n.a.	TMD/OA/M/MP
Kahn, 1998 [67]	US	263	CS		n.a.	TMD/OA/AG
Kirveskari, 1998 [68]	Finland	170	RCT		n.a.	TMD/OA
Manfredini, 2010 [69]	Italia	276	CS	32.2	RDC/TMD	TMD/M
Landi, 2004 [70]	Italia	81	CS	37.2	RDC/TMD	TMD/MP
Ohmori, 2014 [71]	Japan	72	CS	26.6	n.a.	TMD/OA
Pahkala, 2002 [72]	Finland	287	CS	7.6–15.4	HI	TMD/M/OA/AC/MP
Perrotta, 2019 [73]	Italia	700	CS	9–11	n.a.	TMD/M
Pullinger, 1988 [74]	US	222	CS	23.9	n.a.	TMD/OA/M/MP
Pullinger, 1991 [75]	US	106	CS	>21	n.a.	TMD/M
Raustia, 1995 [76]	Finland	49	CS	24	HI	TMD/OA
Riolo, 1987 [77]	US	1342	CS	6–19	n.a.	TMD/OA/AC
Sonnesen, 1998 [78]	Denmark	104	CS	7–13	HI	TMD/OA/M/AC
Nguyen, 2017 [79]	Vietnam	257	CS	65–74	DC/TMD	TMD/E/VDO
Tsolka, 1995 [80]	UK	64	CS	<42	n.a.	TMD/MP
Wang, 2009 [81]	China	741	CS	21–60	NIHTACS	TMD/E/VDO/AG

CS, cross-sectional study approach; RCT, randomized controlled trial; CaG, case group; CtG, control group; TMD, temporomandibular disorder; n.a., not available; FAI, Fonseca Anamnestic Index; RDC/TMD, Research Diagnostic Criteria for TMD; DC/TMD, Diagnostic Criteria for TMD; NIHTACS, National Institutes of Health Technology Assessment Conference Statement; Roc, temporomandibular pain analysis of Rocabado; HI, Helkimo Index; VDO, vertical dimension of occlusion; MP, muscle pain; M, malocclusion; AC, angle class; E, edentulous space; OA, occlusal abnormality; AG, association with gender.

**Table 3:** TMJ involvement correlated with posterior edentulism and VDO changes.

Article	Conclusion
Wang, 2009 [81]	Susceptibility for TMD increases with the following:
	‐ Number of edentulous quadrants
	‐ Number of edentulous spaces
	‐ Increasing age
Joy, 2021 [54]	Severity of TMD increases with the following:
	‐ Decrease in VDO
	‐ Increase in interincisal angle
	‐ Increasing age
Nguyen, 2017 [79]	Subjects with TMD exhibit the following:
	‐ A higher number of posterior edentulous spaces especially in the mandible
Mélou, 2024 [35]	‐ Subjects with untreated edentulous spaces have an increased prevalence of TMD
Malheiros, 2016 [58]	‐ Association between edentulous spaces and TMD

TMD, temporomandibular disorder; VDO, vertical dimension of occlusion.

**Table 4:** The influence of occlusal abnormalities on the temporomandibular joint.

Article	Conclusion
Mélou, 2024 [35]	Increased prevalence of TMD:
	‐ Anterior overlap > 4 mm
	‐ Interferences in laterotrusion
Al-Hadi, 1993 [57]	In occlusions with
	‐ Canine guidance, TMD prevalence is low
	‐ Increased overjet, TMD prevalence is increased
	‐ Interferences on the non-working side, TMD prevalence is increased
Kirveskari, 1998 [68]	‐ Occlusal contacts may play a role in the etiology of TMD
Ohmori, 2013 [71]	‐ Premature occlusal contact is a causative factor of TMD
Pullinger, 1988 [74]	‐ Association between asymmetrical sliding during anterior guidance > 1 mm and joint tenderness
	‐ Association between unilateral premature contact in centric relation with joint tenderness
Raustia, 1995 [76]	‐ Asymmetries of sliding between CR and MI, midline shift, mandible deviation in protrusion, and laterotrusion were associated with signs and symptoms of TMD
Sonnesen, 1998 [78]	‐ Association between TMD and increased overjet, midline shift
Riolo, 1987 [77]	Occlusal abnormalities associated with joint clicking
	‐ Overjet > 6 mm associated with joint clicking
	‐ Cusp to cusp occlusal relationships is a risk factor for joint clicking
	‐ Decreased or increased overjet associated with joint pain
Ozaki, 1990 [61]	Of the subjects with TMD included in the study:
	‐ 42.3%—tooth wear
	‐ 8.5%—bruxism
	‐ 33.8%—unilateral mastication
Seligman, 1989 [59]	‐ Increased MI-CR discrepancy, asymmetric anterior guidance is associated with osteoarthritis
Kahn, 1998 [67]	‐ 33% of subjects—disc displacements without symptoms
	‐ Overbite > 4 mm increases risk for TMD
Barrera-Mora, 2012 [60]	‐ Increased overjet may be a causative factor for TMD
Bindayel N, 2018 [66]	‐ Overjet > 4 mm is a risk factor for TMD
Pahkala, 2002 [72]	Subjects with occlusal interferences showed signs such as
	‐ Clicking
	‐ Deviation of the mandible during opening
	‐ Muscle pain on palpation

TMD, temporomandibular disorder; CR, centric relation; MI, maximum intercuspation.

**Table 5:** The influence of occlusal abnormalities and malocclusions on the temporomandibular joint.

Article	Conclusion
Barrera-Mora, 2011 [60]	‐ Angle class II malocclusion and open bite show the highest prevalence of joints with ligamentous hyperlaxity
	‐ Crossbite may be a causative factor of TMD
Sonnesen, 2008 [63]	‐ Subjects with overbite have bruxism
Bindayel, 2018 [66]	‐ Both psalidodont occlusion and crossbite are risk factors for TMD
Manfredini, 2010 [69]	‐ Overjet > 4 mm, bruxism, and overbite > 5 mm have the highest risk factors for TMD
Pahkala, 2002 [72]	‐ Subjects with angle class III malocclusion are predisposed to mandibular hypomobility
	‐ Subjects with angle class II/1 have a predisposition to mandibular hypermobility
Perrotta, 2019 [73]	‐ There is an association between unilateral/bilateral crossbite, open bite, and TMD
Pullinger, 1988 [74]	‐ Crossbite associated with joint sounds
	‐ Association between angle class II/1 malocclusion and joint tenderness
Pullinger, 1991 [75]	‐ Association between open bite and TMJ osteoarthritis
Seligman, 1989 [59]	‐ Angle class II/1 is more likely to develop TMD than angle class I
	‐ Open bite associated with osteoarthritis
Sonnesen L, 1998 [78]	TMD has been associated with
	‐ Open bite
	‐ Unilateral crossbite
Filho, 2015 [65]	‐ Skeletal class II with open bite is at an increased risk of developing TMD

TMD, temporomandibular disorder; TMJ, temporomandibular joint.

**Table 6:** Relationship between joint impairment and angle class.

Nr	Article	Conclusion
1	Mélou, 2024 [35]	‐ Angle class I has a low prevalence of TMD
2	Sonnesen, 1998 [78]	‐ Association between TMD and angle class II at the molars
3	Riolo, 1987 [77]	‐ Angle class II half cusp has a higher risk of subjects with joint pain, joint sounds, and muscle pain
4	Pahkala, 2002 [72]	‐ Subjects with TMD showed mesialization at the molar level
5	de Paiva Bertoli, 2018 [64]	‐ Angle class II and III show a predisposition for painful symptoms at the myofascial level

TMD, temporomandibular disorder.

**Table 7:** Relationship between masticatory muscle pain and contracture.

Nr	Article	Conclusion
1	Zúñiga-Herrera, 2022 [55]	‐ Oro-facial muscle pain is correlated with and increases in severity with the increasing complexity of dento-maxillary malocclusions, limitation of mouth opening, and depression
2	Ai, 1992 [56]	‐ 96% of subjects with TMD have pain in muscle as well as joint palpation
		‐ Muscle pain shows different patterns depending on the muscle disharmony
		‐ The pterygoid muscles are symptomatic due to their complex anatomy and functions
3	Cooper, 2006 [62]	Muscle pain predominates on palpation:
		‐ Extraoral: anterior fascicle of the temporalis muscle
		‐ Intraoral: lateral pterygoid muscle
4	Sonnesen, 2008 [78]	‐ Subjects with deep bite have muscle pain and arthralgia
5	de Paiva Bertoli, 2018 [64]	‐ Skeletal class II and III have masticatory muscle pain more often than class I
6	Landi, 2004 [70]	‐ CR-MI > 2 mm is associated with muscle pain
7	Pahkala, 2002 [72]	‐ The group of subjects over 15 years of age showed signs such as clicking, muscle spasms of the masticatory muscles, deviation of the mandible during opening
8	Tsolka, 1995 [80]	‐ 39/64 of the subjects with TMD have muscle pain in the masticatory muscles
9	Ozaki, 1990 [61]	Of the TMD subjects in the study
		‐ 64.78% have tenderness on palpation of muscles and TMJ
10	Bindayel, 2018 [66]	‐ Deep bite and crossbite are risk factors for masticatory muscle pain and orofacial pain

TMD, temporomandibular disorder; TMJ, temporomandibular joint; CR, centric relation; MI, maximum intercuspation.

**Table 8:** Degree of joint damage in females.

Nr	Article	Conclusion
1	Kahn, 1998 [67]	‐ Increased prevalence among females
2	Zúñiga-Herrera, 2022 [55]	‐ Females are at higher risk of experiencing muscle pain without limitation of mouth opening
3	Pahkala, 2002 [72]	‐ Female gender was predominantly associated with TMD specific signs and symptoms
4	Wang, 2009 [81]	‐ Female to male ratio affected by TMD is 2:1
5	Ozaki, 1990 [61]	‐ Female to male ratio affected by TMD is 3.4:1
6	Joy, 2019 [54]	‐ Female to male affected by TMD is 20:1
7	Barrera-Mora, 2011 [60]	‐ Females show joint hyperlaxity, deviation during opening, and joint sounds more often than males
8	Cooper, 2006 [62]	‐ The females to male ratio affected by TMD is 3.44:1 in a group of 4528 subjects

TMD, temporomandibular disorder.

## Data Availability

Data are contained within the article.

## Abbreviations

The following abbreviations are used in this manuscript:

O	Observational study
C	Comparative study
CS	Cross-sectional study
CC	Case–control
CaG	Case group
CtG	Control group
TMD	Temporomandibular disorder
VDO	Vertical dimension of occlusion
MP	Muscle pain
M	Malocclusion
AC	Angle class
E	Edentulous space
OA	Occlusal abnormality
AG	Association with gender
TMJ	Temporomandibular joint
CR	Centric relation
MI	Maximum intercuspation
PRISMA-ScR	Preferred Reporting Items for Systematic Reviews and Meta-Analyses extension for Scoping Reviews
